# High-Performance Multiwavelength GaNAs Single Nanowire Lasers

**DOI:** 10.1021/acsnano.3c07980

**Published:** 2024-01-02

**Authors:** Mattias Jansson, Valentyna V. Nosenko, Yuto Torigoe, Kaito Nakama, Mitsuki Yukimune, Akio Higo, Fumitaro Ishikawa, Weimin M. Chen, Irina A. Buyanova

**Affiliations:** † Department of Physics, Chemistry and Biology, Linköping University, SE-58183 Linköping, Sweden; ‡ Graduate School of Science and Engineering, Ehime University, Matsuyama 790-8577, Japan; § Research Center for Integrated Quantum Electronics, Hokkaido University, Sapporo 060-8628, Japan; ∥ Systems Design Lab (d.lab), School of Engineering, The University of Tokyo, Tokyo 113-8656, Japan

**Keywords:** nanowires, lasing, nonlinear optics, nanophotonics, coherent light, second harmonic generation (SHG), multiwavelength coherent light

## Abstract

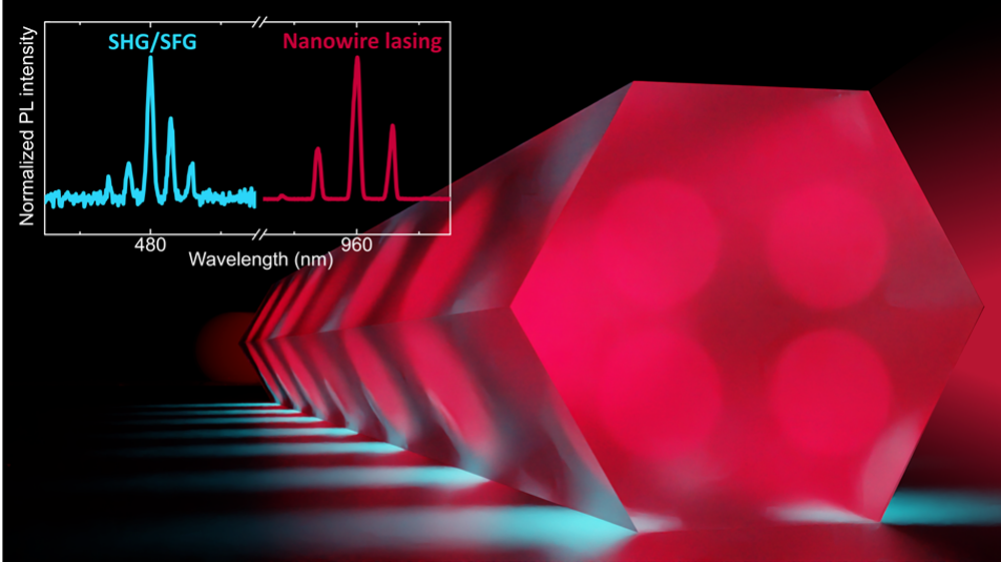

In this study, we report a significant enhancement in the performance of GaNAs-based single nanowire lasers through optimization of growth conditions, leading to a lower lasing threshold and higher operation temperatures. Our analysis reveals that these improvements in the laser performance can be attributed to a decrease in the density of localized states within the material. Furthermore, we demonstrate that owing to their excellent nonlinear optical properties, these nanowires support self-frequency conversion of the stimulated emission through second harmonic generation (SHG) and sum-frequency generation (SFG), providing coherent light emission in the cyan-green range. Mode-specific differences in the self-conversion efficiency are revealed and explained by differences in the light extraction efficiency of the converted light caused by the electric field distribution of the fundamental modes. Our work, therefore, facilitates the design and development of multiwavelength coherent light generation and higher-temperature operation of GaNAs nanowire lasers, which will be useful in the fields of optical communications, sensing, and nanophotonics.

The development of efficient and versatile nanoscale lasers has garnered considerable attention due to their potential to revolutionize a wide array of applications, ranging from optical communications^1^ and photonic integrated circuits,^2^ to sensing,^3^ metrology^4^ and microscopy.^5,6^ Semiconductor nanowires (NWs) represent a particularly promising platform for the development of nanoscale lasers, as they inherently combine an active gain medium with a Fabry–Perot cavity.^7,8^ Moreover, the seamless integration of semiconductor NWs with silicon enables the fusion of nanoscale photonics and microelectronics.^9−11^ In addition to lasing at a single wavelength, semiconductor NWs allow the realization of multicolor lasing with a large spectral separation between the lasing wavelengths, expanding the range of potential applications to color displays and optical parametric generators. For these purposes, the wavelength tunability due to a compositional gradient of the gain medium, e.g., from II–VI or perovskite semiconductor alloys, was mainly explored,^12,13^ though self-frequency conversion of the lasing emission via nonlinear processes was also most recently demonstrated in InGaAs NWs.^14^

A crucial task in designing NW lasers is to find materials where the gain spectral range matches the desired lasing wavelength, which is not always an easy task, depending on the wavelength range in question. For example, near-infrared (NIR) lasers based on InGaAs/GaAs heterostructured NWs may suffer from a high compressive strain resulting in plastic deformation.^1^ A promising alternative material for wavelength tuning within the NIR spectral range is GaNAs, where a small fraction of incorporated nitrogen gives rise to a huge down-shift of the conduction band edge, owing to the giant bandgap bowing effect in this material.^15^ For instance, by incorporating only 2% of nitrogen in GaAs/GaNAs NW heterostructures lasing at 1 μm was achieved,^16^ approaching optical communication wavelengths. Unfortunately, to date GaNAs NW lasers suffer from a high lasing threshold (>15 μJ/cm^2^/pulse) and a low maximum operation temperature (100 K).^16,17^ To fully harness their potential, it is imperative to enhance the NW performance by minimizing the lasing threshold, thereby facilitating lasing at higher temperatures.

In this work, we present a significant improvement in the growth of GaAs/GaNAs/GaAs core/shell/cap NW lasers by employing selective area epitaxy (SAE), leading to substantial enhancements of their performance in terms of both threshold power and operation temperature compared to the best GaNAs-based NW lasers demonstrated to date. To further assess the potential of GaNAs-based nanolasers for optoelectronic applications, we explore nonlinear optical phenomena in the NWs under lasing conditions, which have not previously been investigated for this material system. We reveal self-frequency conversion of the fundamental laser light through second harmonic generation (SHG) and sum-frequency generation (SFG). This enables coherent light emission in the cyan-green range around 500 nm, which has in the past proven difficult to achieve using, e.g., InGaN NWs.^14,18^ We compare the self-conversion efficiency between different fundamental lasing modes and identify the mode-specific differences by simulating the electric field distribution and analyzing the nonlinear susceptibility coefficients. Our work, therefore, not only significantly advances the performance of GaNAs-based NW lasers but also deepens our understanding of the underlying nonlinear optical phenomena. These breakthroughs facilitate the development of multiwavelength coherent light generation and higher-temperature operation of GaNAs NW lasers, which will be of benefit in the fields of optical communication, sensing, and nanophotonics.

## Results and Discussion

### Growth

Previously, the growth of optically pumped GaNAs-based NW lasers using plasma-assisted molecular beam epitaxy (MBE) has been performed on epi-ready Si(111) substrates that were not subjected to any treatment. Therefore, nucleation of NWs, which were self-catalyzed by supplied gallium (Ga) forming droplets, occurred at pinholes of a thin native oxide that covered the substrates. The growth was performed in an MBE system, which was equipped with a water-cooled shroud with a background pressure of about 2 × 10^–9^ Torr, at a V/III flux ratio of 1.^19,20^ In order to improve the performance of the NW lasers, in this study, we used a different growth method. First of all, the MBE chamber was now cooled with liquid nitrogen, which decreased its background pressure to 5 × 10^–10^ Torr, important for suppressing incorporation of background impurities in the grown layers. Moreover, the growth was performed on patterned Si substrates with predefined openings in the SiO_2_ surface layer, as such SAE enables nucleation of the NWs over a wider range of growth parameters.^21,22^ In addition, in order to improve the structural uniformity of the NWs, we also increased the V/III ratio to 3. According to previous studies,^23−28^ a high V/III ratio improves crystallographic properties of III–V NWs by suppressing formation of planar structural defects, such as rotational twins. It should also ensure a uniform nitrogen distribution during the vapor–solid growth of the GaNAs shell, similar to the case of thin film growth.^29^ A detailed description of the growth conditions can be found in the Experimental Section.

### Structural Properties

**Figure 1:**
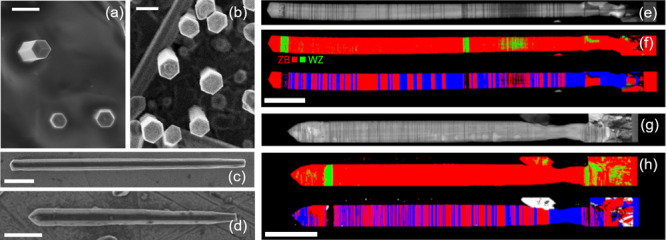
(a–d) Top-view SEM images of as-grown standing SAE-grown (a) and reference (b) NWs, and their corresponding lying NWs transferred to gold substrates (c, d, respectively). (e, g) TEM images of the SAE-grown (e) and reference (g) NWs. (f, h) IPF maps resolving the phase of ZB and WZ segments (denoted as the red and green segments in the upper images) and the orientation of ZB segments (the blue and red segments in the lower images) of a SAE-grown (e) and reference (h) NW. The color switch observed for the IPF map corresponds to the existence of twin defects. The scale bars in (a–b) and (c–h) are 300 nm and 1 μm, respectively.

Using the SAE growth method, we fabricated radial GaAs/GaNAs/GaAs heterostructured NWs and compared their performance with reference structures with nominally the same layer thicknesses and N composition but grown using the previously used technique.^19^ Scanning electron microscopy (SEM) images of standing NWs can be seen in Figure 1a,b for the SAE-grown and reference samples, respectively, while the corresponding SEM images of lying NWs transferred to a gold substrate are shown in Figure 1c,d. We see that both growth techniques produce NWs with well-defined hexagonal cross sections and similar geometries, though SAE-grown NWs tend to be longer, averaging 7.7 ± 1.4 μm as compared with 4.2 ± 1.1 μm for the reference NWs (the uncertainty range is the standard deviation among the 97 NWs investigated by SEM). Both types of NWs have similar cross-sectional diameters ranging between 350 and 400 nm. According to the performed transmission electron microscopy (TEM) measurements, both types of NWs have a predominantly zinc-blende (ZB) lattice structure with minor wurtzite (WZ) inclusions. However, the SAE-grown structures contain a lower number of rotational twin planes (seen as dark lines in the TEM images shown in Figure 1e,g). This is further apparent from Figure 1f,h, which show inverse pole figure (IPF) maps resolving the ZB and WZ segments (the red and green areas in the upper image) and the orientation of ZB segments (the blue and red areas in the lower image) for the SAE-grown and reference NW, respectively, obtained by crystal diffraction mapping.^30−34^ The lattice direction is opposite between the switching interfaces in the ZB segments, indicated by blue and red in the orientation map. This indicates the existence of twin defects at the switching interfaces, which correspond to the positions of the dark lines observed in the TEM images. The lower density of structural defects in the SAE-grown NWs is likely caused by a higher V/III ratio during the growth, consistent with the previous studies.^23−28^

**Figure 2:**
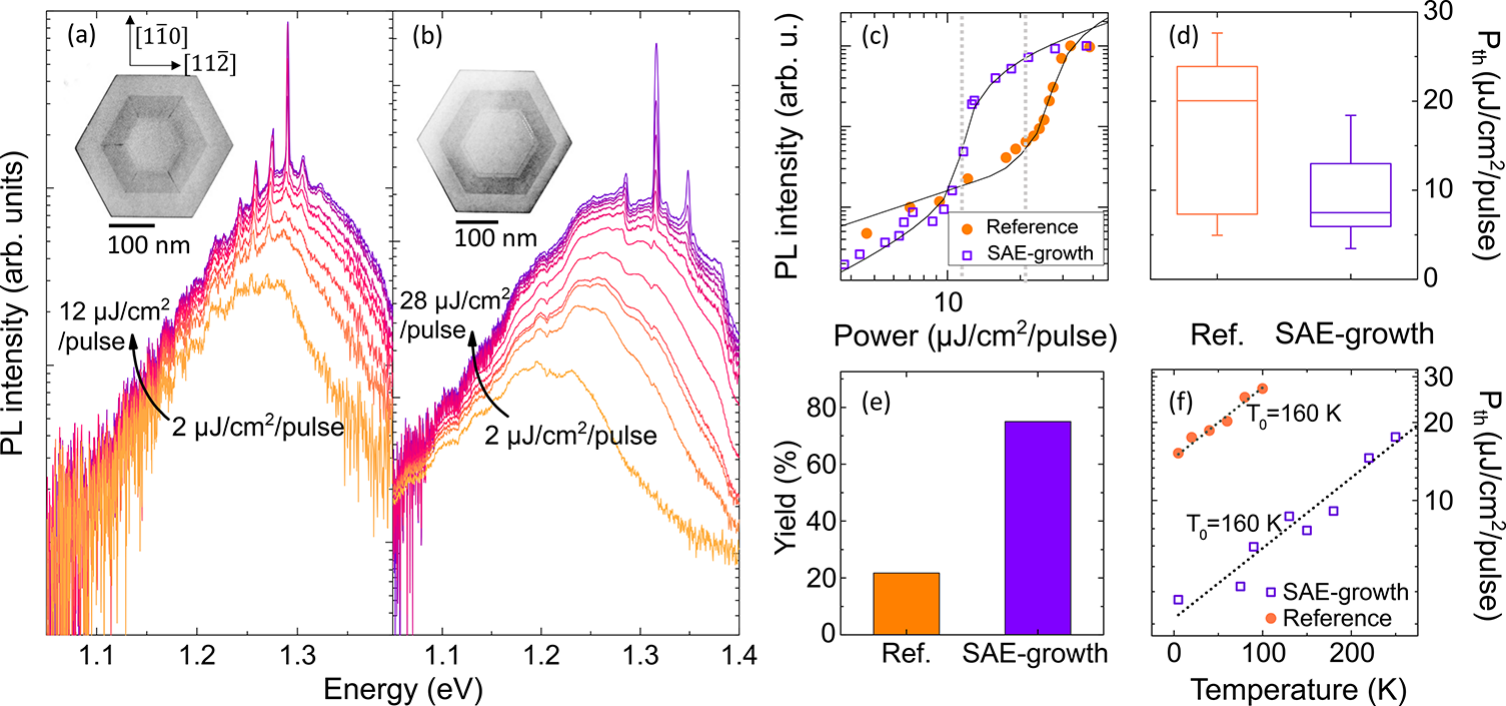
(a, b) Power-dependent PL spectra acquired at 5 K from an SAE-grown (a) and reference (b) NW of lengths of 8.0 and 6.6 μm, respectively, using pulsed excitation at 800 nm. The insets show cross-sectional BF-STEM micrographs of representative NWs from the two structures. (c) Measured PL intensity of the lasing modes in the SAE-grown (the purple squares) and reference (the orange circles) NW, and simulated photon density using the rate equation analysis described in Supporting Information, section 8. (d) Box-plot showing the lasing threshold power measured from 50 NWs, where the box represents the 25 and 75 percentiles. (e) The yield: percentage of the investigated NWs which exhibit lasing. (f) Temperature dependence of the lasing threshold of an SAE-grown (the purple squares) and reference (the orange circles) NW. The dotted lines represent the best fit to the experimental data using the equation *P*_th_ ∝ *e*^*T*/*T*_0_^, where *P*_th_ is the lasing threshold power, and *T*_0_ is the characteristic temperature, which is deduced to be 160 K for both samples.

Typical cross-sectional scanning TEM (STEM) images of a single SAE-grown and reference NW are shown in the insets of Figure 2, panels a and b, respectively. The STEM results confirm the intended core/shell/cap structure with the 120–190 nm thick GaAs core, the 40–50 nm thick GaNAs shell (the darker area in the STEM images), and the 30–50 nm thick GaAs capping layer.

### Lasing Performance

In order to assess the lasing performance of the NWs, we measured their photoluminescence (PL) spectra using pulsed laser excitation. In both structures the PL spectra (shown in Figure 2a,b) transform from a broad emission, which dominates at low pumping powers (*P*_exc_), to a series of very sharp lines in the wavelength range of 900–1000 nm characteristic of lasing from the intrinsic Fabry–Perot cavity of the NWs defined by their end facets. The transition from spontaneous emission to lasing is further confirmed by the apparent S-shape dependence of the PL intensity, shown in Figure 2c. By analyzing the mode spacing between the lasing peaks of the NWs with different lengths, as well as the polarization patterns of the lasing emission, we conclude that the detected lasing in the majority of NWs originates from the fundamental HE_11a/b_ modes though lasing via the HE_21b_ mode can also be observed (see sections S2 and S3 of the Supporting Information). This is expected, as according to performed finite-difference time-domain (FDTD) simulations, these modes have the lowest threshold gain values for wavelengths exceeding 950 nm. Interestingly, the SAE-grown NWs exhibit two noteworthy improvements in the lasing performance. First, a reduced average lasing threshold among lasing NWs, from 21.4 to 6.9 μJ/cm^2^/pulse (Figure 2d), is found from a set of 50 NWs. By performing a statistical two-sample *t* test analysis of the data,^35^ we find with *p* < 5% (*p* = 5 × 10^–5^) a significant difference between the lasing threshold of the two samples. Second, a higher yield (75%) of lasing NWs as compared with 22% in the reference structures (Figure 2e) is found by investigating 140 NWs. Furthermore, SAE growth results in a narrower range of lasing threshold values, indicating increased uniformity among the NWs. The reduced lasing threshold significantly improves the temperature limit for lasing operation, as shown in Figure 2f: the SAE-grown structures sustain lasing up to 250 K, a substantial increase as compared with 100 K in the reference structures. By fitting the measured temperature dependence of the threshold power, *P*_th_, by the function *P*_th_ ∝ *e*^*T*/*T*_0_^, a high characteristic temperature of *T*_0_*=* 160 K can be deduced for both structures.

In principle, the improved lasing performance in the SAE-grown NWs could originate from better material quality, such as a reduced rate of competing nonradiative recombination. However, the NW brightness at low temperatures and activation of nonradiative recombination channels at elevated temperatures are strikingly similar between the two structures (see section S4 of the Supporting Information). Additionally, slight variations in the NW geometry of the SAE and reference structures (e.g., widths and lengths) do not appear to strongly affect the lasing threshold (see section S5 of the Supporting Information) and certainly cannot account for the large difference in its value between the SAE-grown and reference structures. To explain the observed improvements in the lasing performance due to the SAE growth method, we draw attention to the power-dependence of the PL spectra in Figure 2a,b. The reference NW (Figure 2b) exhibits a substantial blue-shift of the emission peak with increasing excitation power, indicating a significant degree of exciton localization within the band-tail states characteristic of dilute nitrides. Conversely, the SAE-grown NW demonstrates a notably smaller blue-shift, suggesting a considerably lower concentration of localized states in this structure. We note that a strong localization is characteristic for GaNAs alloys, where minor fluctuations in the N content are known to cause significant changes in the conduction band edge due to the strong bandgap bowing effect.^36^ In NWs, localization should be further enhanced by structural polytypism due to differences in the electronic structure of ZB and WZ GaAs.^37^ Since the influence of localized states on the lasing performance of NWs has not been examined previously, we conduct a thorough analysis of their impact.

**Figure 3:**
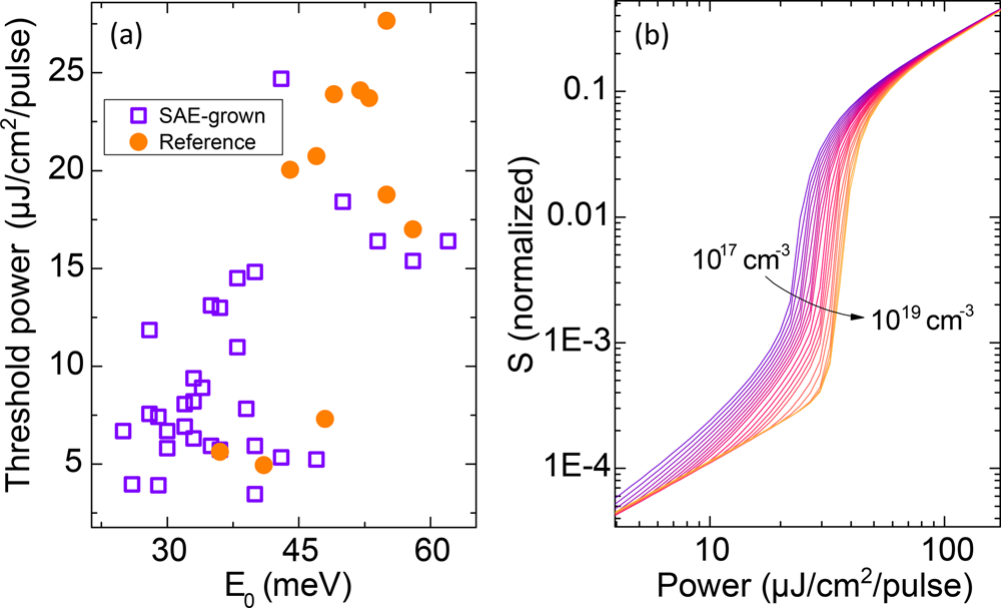
(a) The measured threshold power, *P*_th_, vs the localization energy, *E*_0_, deduced from the PL spectra of SAE-grown (the purple squares) and reference (the orange circles) NWs, respectively. The measurements were performed at 5 K. (b) Simulated photon density, *S*, as a function of excitation power density, using the rate equations described in section 7 of the Supporting Information, with β = 0.05 and varying the density of localized states, *D*_LS_ between 10^17^ (the dark purple line) and 10^19^ (the bright orange line) cm^–3^.

To determine the degree of localization in the NWs, we adopted a measure based on the localization energy, *E*_0_. While directly gauging the density of localized states in a NW is challenging, *E*_0_ offers a feasible alternative because it can be inferred from the low energy tail of the PL spectrum (for details, refer to section S6 of the Supporting Information). When we compare the lasing threshold power with *E*_0_ (Figure 3a), a positive linear correlation emerges at the 5% significance level (with ρ = 0.69 and *p* = 5 × 10^–7^); see also section S9 of the Supporting Information. This suggests that variations in the threshold power among the NWs are notably influenced by the fluctuations in *E*_0_. However, the relatively low *R*^2^ value (*R*^2^ = 0.48) shows that the variation in threshold power is affected by other parameters as well, such as the end facet geometry and nanowire length,^38^ though the latter is not a dominant factor in the studied NWs; see section S5 of the Supporting Information.

To further illustrate the influence of exciton localization on the lasing threshold, we implemented a rate-equation analysis (detailed in section S7 of the Supporting Information), to model the photon density (*S*) as a function of the excitation power for varying localized state density. Results from these simulations, shown in Figure 3b, indicate a marked shift in the lasing threshold as the density of localized states changes. Moreover, our rate-equation model successfully reproduces the power-dependence of the PL intensity (shown in Figure 2c by the solid lines) using identical simulation parameters for the SAE-grown and reference samples, except for a notable increase in the density of localized states from 6 × 10^16^ to 7 × 10^18^ cm^–3^. This accentuates the crucial role of exciton localization in the NW lasing performance. Additionally, the spontaneous emission coupling factor, β, is 0.010 for the SAE grown sample and 0.044 for the reference sample. We note that, for the simulation parameters used in this study, this change in β does not impact the lasing threshold (see Supporting Information, section S10 for details). These results underline the significant influence of the exciton localization on the NW lasing performance, important for designing NW lasers using highly mismatched materials.

### Self-Frequency Conversion

**Figure 4:**
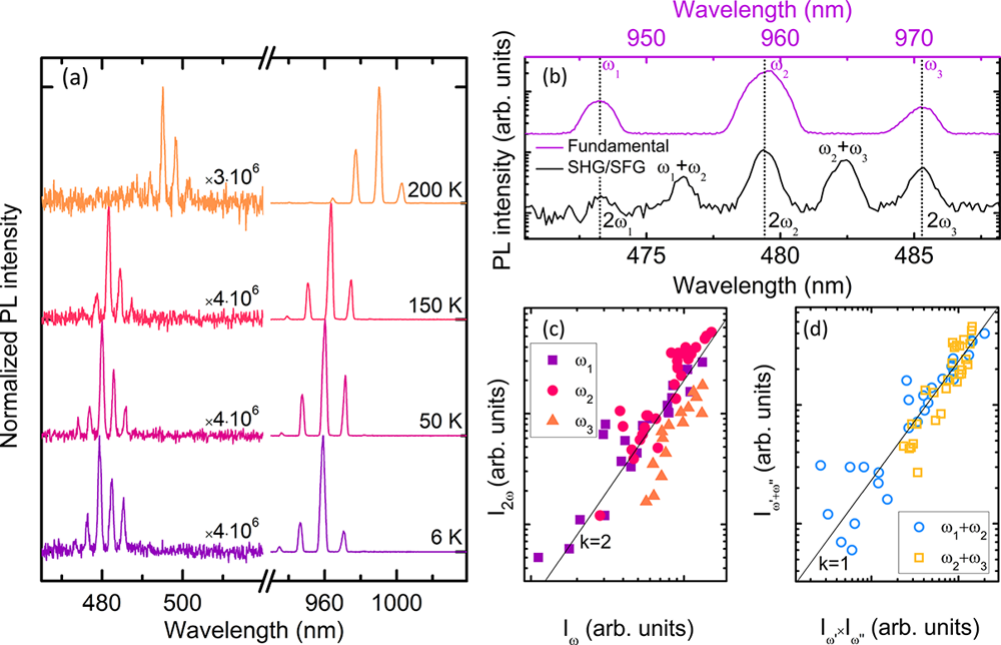
(a) PL spectra acquired from a SAE-grown NW under lasing conditions at different temperatures, showing emissions in both the NIR and visible spectral ranges. The visible range spectra have been normalized to those in the NIR range using the scaling factors displayed in the figure. (b) Magnification of the 6 K spectra from (a). (c, d) The intensity of the SHG and SFG peaks as a function of the corresponding fundamental lasing peak intensity (c) or the product of the two corresponding fundamental lasing peak intensities (d). The solid lines illustrate quadratic (c) and linear (d) dependence.

Most recently, self-frequency conversion of the lasing emission via nonlinear processes has been demonstrated in InGaAs NWs, extending the spectral range of the NW lasers beyond that determined by the material gain of the employed semiconductor.^14^ To establish the importance of such processes in dilute nitride NWs, we examine the PL spectra in the visible spectral range during lasing conditions. We observe multiple sharp lines within the blue-green spectral range (Figure 4a), which indicates involvement of nonlinear optical processes resulting in photon upconversion. Some of these high-energy lines appear at precisely half the wavelength of the fundamental lasing (Figure 4b), suggesting that they are the result of second harmonic generation (SHG) of the fundamental lasing light emitted by the NW. This assignment is further supported by the quadratic power dependence of the SHG signal on the intensity of the fundamental lasing light; see Figure 4c. The upconverted spectra also contain additional peaks located between the SHG peaks, which arise from the sum frequency generation (SFG) of two fundamental mode peaks due to a three-wave-mixing process. The intensity of these peaks, *I*_ω′+ω″_, varies approximately linearly with the product of the two constituent fundamental mode intensities *I*_ω′_ and *I*_ω″_ (Figure 4d), as expected for the SFG process.^39^ The upconverted emission can be observed as long as the fundamental lasing is achieved, e.g., at 200 K as shown in Figure 4a, which is significantly higher than the previously reported temperature of 10 K for self-frequency-conversion in InGaAs NWs^14^ and is limited by the lasing threshold of the fundamental lasing in the studied NWs. Moreover, the near-constant generation efficiency across this temperature range (as seen from the scaling factors in Figure 4a) is attractive for practical applications.

**Figure 5:**
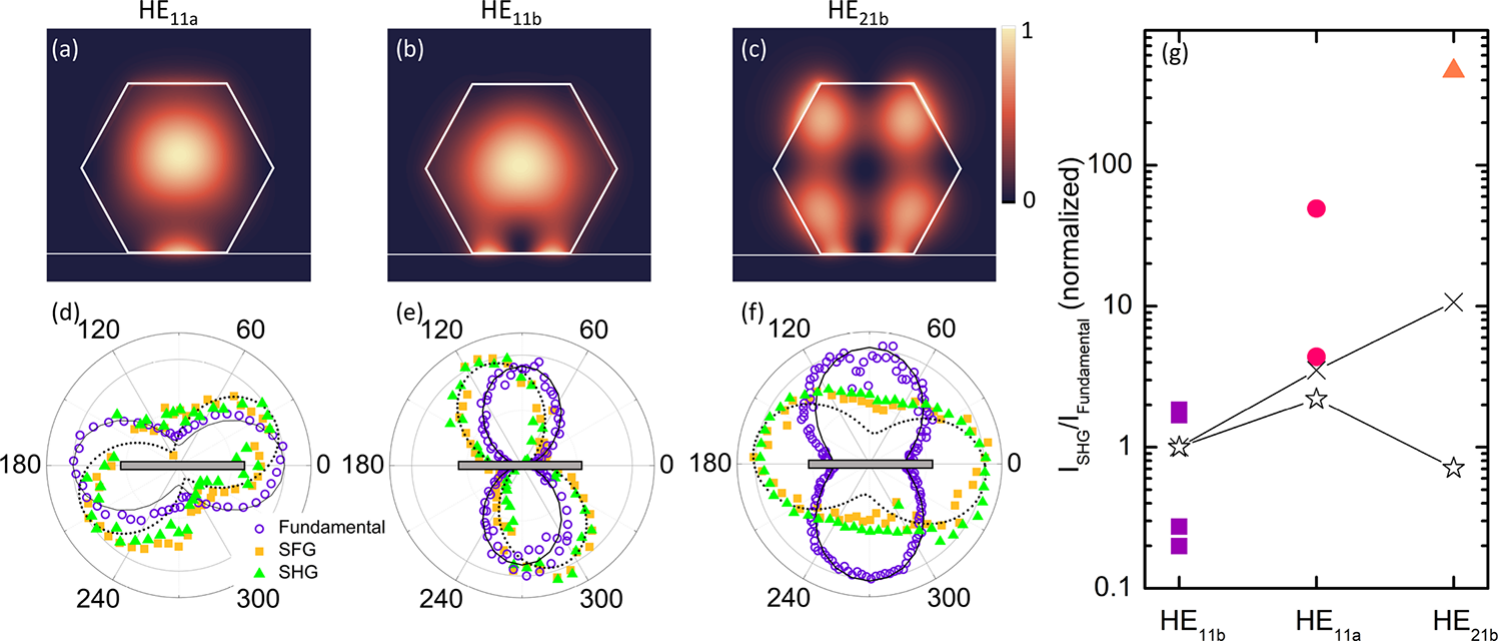
(a–c) Simulated |*E*|^2^ field distribution for the HE_11a_, HE_11b_, and HE_21b_ modes, respectively. (d–f) Measured polar plots of the fundamental modes (the purple circles), the SFG (the yellow squares), and SHG (the green triangles) emission from three different NWs. The solid lines represent the simulated intensity distributions of the fundamental modes (HE_11a_, HE_11b_, and HE_21b_ respectively) deduced from the FDTD calculations, whereas the dotted lines show the expected polarization pattern of the corresponding upconverted SHG and SFG signals. (g) The intensity ratio of the upconverted (SHG + SFG) and fundamental lasing light measured from different NWs exhibiting the HE_11a_, HE_11b_, and HE_21b_ mode lasing (the symbols). The data were normalized to the mean value of the HE_11b_ mode intensity. The stars show the expected ratios for the three different modes, taking into account eq 1, while the crosses show the calculated ratios using eq 1 combined with FDTD simulations of the light extraction efficiency. The solid lines are guides for the eye for the simulated values.

We have established (see section S3 of the Supporting Information) that multiple lasing modes are observed in the studied NWs, including the HE_11a_, HE_11b_, and HE_21b_ modes. Their intensity distributions within the NW modeled using a finite-difference-time-domain (FDTD) calculations are shown in Figure 5, panels a–c, respectively. With these modes identified, it is interesting to examine their nonlinear optical response, and it is important for optimizing the self-frequency-upconversion process. For these purposes we first compared the polarization properties of the fundamental modes, and their corresponding nonlinear components (Figure 5d–f). As expected,^8^ the HE_11a_ fundamental lasing light (the purple circles) is polarized parallel to the long axis of the NW (represented by the gray bar in Figure 5d–f), while the HE_11b_ and HE_21b_ modes have polarization orthogonal to the NW axis. This agrees with the results of the FDTD calculations shown by the solid lines. On the other hand, according to the polar plots of the SFG and SHG signals (the orange and green symbols, respectively), their polarization deviates from both the axial [111] and radial [112̅] directions. Moreover, the polarization pattern of the upconverted light is found to be unique for each fundamental lasing mode, suggesting that it can be tailored by choosing the dominant lasing mode. To corroborate this experimental finding, we calculated the expected polarization pattern of the SHG and SFG signals. The SHG response in the crystal frame, *c*, is based on the second-order polarization *P*_c_. Since ZB GaAs is a noncentrosymmetric crystal belonging to the 4̅3*m* crystal symmetry group, its second-order nonlinear susceptibility tensor d^(2)^ has three nonvanishing elements *d*_14_ = *d*_25_ = *d*_36_ = 370 pm/V.^14^ Therefore, *P*_c_ is given by
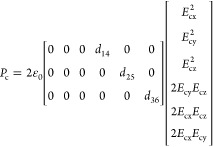
1Here ε_0_ is the vacuum permittivity, and *E*_c*i*_ are the electric field components in the *i* crystal direction defined within the crystal frame. By simulating the electric field distribution of the three observed fundamental modes using an FDTD algorithm and rotating the calculated *P*_c_ to the lab frame, we can compute the expected polarization pattern of the upconverted light using eq 1 (see the dotted lines in Figure 5d–f). Since the exact values of the d^(2)^ elements in GaNAs is still unknown, we use the same values of the d^(2)^ tensor in both GaAs and GaNAs materials as an approximation. The close agreement between the calculated and measured polarization patterns of the upconverted signals supports the mode assignment and confirms the origin of the upconverted emission.

The efficiency of frequency conversion is paramount for any technological implementation of nonlinear phenomena. By evaluation of the upconversion efficiency, which was measured here as the ratio between the upconverted (SHG + SFG) and fundamental light intensities, a pronounced disparity among modes becomes evident (see Figure 5g). Specifically, the measured upconversion efficiency is the lowest for the HE_11b_ mode (squares), is somewhat higher for the HE_11a_ mode (dots), and increases by more than 100 times for the HE_21b_ mode (triangles). Calculating the relative upconversion strength of the different modes using eq 1 (shown by the stars in Figure 5g), we find that although the upconversion efficiency for the HE_11a_ mode is indeed higher than that of the HE_11b_ mode, as seen from the measurements, it is greatly underestimated for the HE_21b_ mode. To understand this discrepancy between the calculated and experimental values, we note that the upconverted photon energy exceeds the GaAs and GaNAs band gap energies, resulting in a substantial absorption of the generated SHG/SFG light. Comparing the distributions of the electric field (***E***) in Figure 5a–c, it is clear that the electric field of the HE_21b_ mode is distributed closer to the NW surface as compared with the HE_11a_ and HE_11b_ modes, which should result in decreased absorption of the corresponding upconverted light. To quantitatively investigate this effect, we perform FDTD simulations to calculate the far-field intensity of the electric field produced by a source corresponding to the SHG light calculated using eq 1 for the three modes. By taking into account this effect, simulations indeed suggest the highest upconversion efficiency for the HE_21b_ mode, albeit lower than the experimentally determined value (see the crosses in Figure 5g). Several reasons could be responsible for this underestimation of the SHG efficiency for the HE_21b_ mode. First of all, the electric field of the HE_21b_ mode overlaps considerably with that of the GaNAs shell, whereas the HE_11a_ and HE_11b_ modes are mostly confined to the GaAs core. Past studies have shown that dilute nitride materials, like GaNAs, exhibit a higher SHG efficiency compared to their nitrogen-free counterparts.^40,41^ This suggests that we could expect a higher SHG generation efficiency for the HE_21b_ mode relative to that for the HE_11a_ and HE_11b_ modes. Another possibility is a different strain distribution in the shell layers (where the HE_21b_ mode is primarily confined) compared to the core region (hosting the HE_11a_ and HE_11b_ modes), which will further influence the nonlinear susceptibility tensor. Further studies are required to clarify this important result.

We note that in addition to the SHG and SFG, another intriguing aspect of the observed self-frequency conversion in the studied NWs is its potential for difference frequency generation (DFG) of the fundamental lasing light. This process involves an energy downconversion, producing a photon with energy equal to the energy difference between two fundamental photons. For instance, referencing the spectrum in Figure 4b, the anticipated DFG energy is around 17 meV. This falls within the terahertz range, implying that GaNAs NWs undergoing self-frequency conversion could serve as a coherent THz source. While our current experimental setup does not allow detection of such THz signals, it is worth noting that both SFG and DFG are second-order processes as outlined by eq 1. Hence, they are predicted to happen with equivalent efficiency, suggesting that the DFG emission is likely present in the examined NWs.

## Conclusions

In conclusion, we have successfully improved the growth conditions of GaNAs NW lasers, resulting in a significant enhancement in their performance. The new growth method, based on selective-area epitaxy on electron-beam-patterned substrates, led to a higher yield of lasing NWs, a reduced average lasing threshold, and an increased uniformity of the lasing characteristics among the NWs. Notably, the improved structures demonstrated lasing up to 250 K, a substantial increase compared with the best previously reported GaNAs NW lasers. Through a detailed investigation, we have attributed this improvement to a reduced density of localized states in the GaNAs alloy achieved under the SAE-growth. Furthermore, our study has explored the nonlinear optical phenomena occurring in GaNAs NWs under lasing conditions, demonstrating self-frequency conversion through SHG and SFG. The measured SHG/SFG efficiency was found to differ significantly between different fundamental lasing modes, which could be attributed to combined effects of the electric field distribution of the fundamental modes, their different light extraction efficiencies, as well as an improved nonlinear response of the GaNAs alloys as compared with parental GaAs. These advancements in the NW laser performance and the understanding of nonlinear optical phenomena facilitate the development of multiwavelength coherent light generation and room temperature applications of GaNAs-based NW lasers.

## Experimental Section

### Materials

The investigated samples were grown in a plasma-assisted molecular beam epitaxy (MBE) system.^19,20^ For the SAE-growth, we used a patterned substrate, which was fabricated by creating square openings of 300 × 300 nm^2^ in a SiO_2_-covered n-type Si(111) wafer. The template was prepared by sputtering, electron beam lithography, and inductively coupled plasma reactive ion etching.^42^ For the MBE growth, a conventional solid-source effusion cell was used for the supply of Ga, whereas As was supplied by using an As-valved cracker cell operating in the As_4_ mode. Nitrogen was supplied by a radio frequency plasma source. The GaAs NW core was then formed by vapor–liquid–solid growth assisted by constituent Ga seed particles when Ga and As fluxes were supplied on the substrate.^19,20^ The beam equivalent pressure (BEP) of As_4_ was set to 6 × 10^–4^ Pa throughout the growth, corresponding to the growth rate of 1.0 monolayer (ML)/s on GaAs(001). The atomic V/III flux ratio was estimated from the reflection high energy electron diffraction of the GaAs thin film grown on GaAs(001) substrate^43^ based on their intensity oscillation and transition point between Ga rich and As rich patterns.^44^ The Ga BEP was 3 × 10^–5^ Pa, and the flux was set to match a planar growth rate of 0.3 ML/s on GaAs(001) throughout the growth. The V/III ratio was thus 3 under these conditions. The GaAs core growth was performed in two steps. First, the growth was initiated by opening the Ga shutter under As overpressure. The GaAs core was then grown for 30 min at 560 °C. When a growth interruption was introduced, the catalyst Ga became crystallized. Then the growth of the GaAs core was finalized. The first GaAs shell was grown for 20 min, followed by a second growth interruption. Subsequently, the lateral growth became dominant, and we completed the growth of GaAs core for 30 min, followed by the second growth interruption. During the interruption, the growth temperature was reduced to 500 °C, and the nitrogen plasma was ignited. By opening the shutter of the plasma source, the GaNAs shell was grown, which was followed by the fabrication of the outermost GaAs shell, forming a GaAs/GaNAs/GaAs core–multishell structure.^19,20^ The reference NWs were grown using a similar process, but the NWs were grown on an untreated n-type Si(111) substrate in an MBE system with a water-cooled shroud, using a V/III flux ratio of 1. Details on the reference sample growth can be found elsewhere.^19^ Both SAE-grown and reference NWs have very similar N compositions of 2.2 and 2.3%, respectively, as estimated based on PL excitation measurements; see section S8 of the Supporting Information.

### Methods

Structural characterization of the NWs was carried out using SEM (Zeiss Sigma 300 SEM) with an extraction voltage of 2–4 kV and TEM. Axially and radially sliced single NW samples were prepared by focused ion beam processing (Helios660, FEI). Axial cross-sectional structural investigations were carried out with STEM (JEM-2100F from JEOL at 200 kV). Radial cross-sectional electron diffraction mapping of the nanowires was performed using STEM (JEM-ARM200F Dual-X TEM microscope from JEOL at 200 kV) equipped with a scanning precession electron diffraction system (ASTAR, NanoMEGAS) of an automated crystal orientation mapping technique.^29−34^ To optically characterize individual NWs, we mechanically transferred them to either SiO_2_ or Au substrates. The transfer process resulted in a NW separation generally exceeding 10 μm, allowing single NW spectroscopy. Optical characterization of individual NWs was carried out using a μPL setup, where the excitation light was focused using a 50× 0.5 NA objective lens on individual NWs on SiO_2_ or Au substrates, mounted in an Oxford Instruments Microstat HiRes cryostat operating in the temperature range of 5–300 K. The PL signal was collected in the backscattering geometry by the same objective lens and subsequently filtered by appropriate long-pass or short-pass filters for fundamental or upconverted lasing light detection, respectively. The emitted light was then dispersed by using a single-grating monochromator and detected by using either an LN_2_-cooled InGaAs linear array detector (for NIR light) or a Peltier-cooled Si CCD detector (for visible-light). Polarization-resolved measurements were carried out in the same setup, employing a rotatable half-wave plate in combination with a stationary linear polarizer positioned before the monochromator entrance. As an excitation source, we used a tunable Ti:sapphire laser operating in either pulsed (with a pulse width of 150 fs and a repetition rate of 76 MHz) or continuous-wave mode. The excitation wavelength was 800 nm for all experiments.

## Supporting Information Available

Structural characteristics. Threshold gain calculations. Mode analysis. Optical quality of the NWs based on temperature-dependent PL measurements. Effects of the structural properties on the lasing threshold. Estimates for localization energy. Rate equation analysis. Bandgap energy estimation. Statistical analysis (PDF)
