# Supplementary material for: High-Performance Multiwavelength GaNAs Single Nanowire Lasers
Source: ACS Nano. 2024 Jan 2;18(2):1477–84. doi: 10.1021/acsnano.3c07980 (PMC10795468; doi:10.1021/acsnano.3c07980)
Supplement: Supplementary file 1 — nn3c07980_si_001.pdf [file nn3c07980_si_001.pdf]

Supporting information

High-Performance Multi-Wavelength GaNAs Single Nanowire Lasers

Mattias Jansson[1], Valentyna V. Nosenko[1], Yuto Torigoe,[2] Kaito Nakama,[3] Mitsuki Yukimune,[2] Akio Higo,[4] Fumitaro Ishikawa[3*], Weimin M. Chen[1], Irina A. Buyanova[1*]*

Email: mattias.jansson@liu.se

Email: irina.bouianova@liu.se

Email: ishikawa.fumitaro@rciqe.hokudai.ac.jp

[1]Department of Physics, Chemistry and Biology, Linköping University, SE-58183 Linköping, Sweden

[2]Graduate School of Science and Engineering, Ehime University, Matsuyama 790-8577, Japan

[3]Research Center for Integrated Quantum Electronics, Hokkaido University, Sapporo 060-8628, Japan

[4]Systems Design Lab (d.lab), School of Engineering, The University of Tokyo, Tokyo 113-8656, Japan

S1. Structural characteristics

Figures S1 shows representative scanning electron microscopy (SEM) images of several nanowires (NW) grown by selective area epitaxy (SAE) (a-c) and reference (d-f) structures, transferred to gold substrates. Figure S2 similarly shows top-view SEM images of as-grown NWs grown by the SAE and reference growth methods. From the top-view SEM images, undergrowth on the substrate can be clearly observed in the reference sample (Figure S2b), while the substrate of the SAE-grown NWs does not show evidence of such undergrowth deposition.

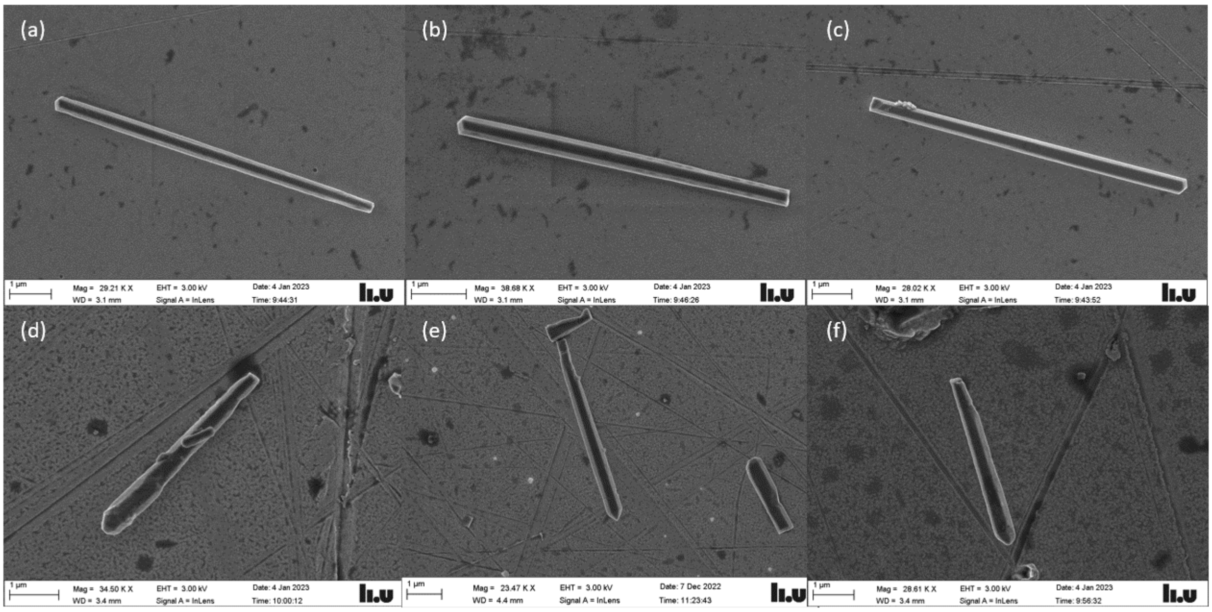

Figure S1: SEM micrograph of NWs grown using the SAE (a-c) and reference (d-f) growth methods, transferred to gold substrates.

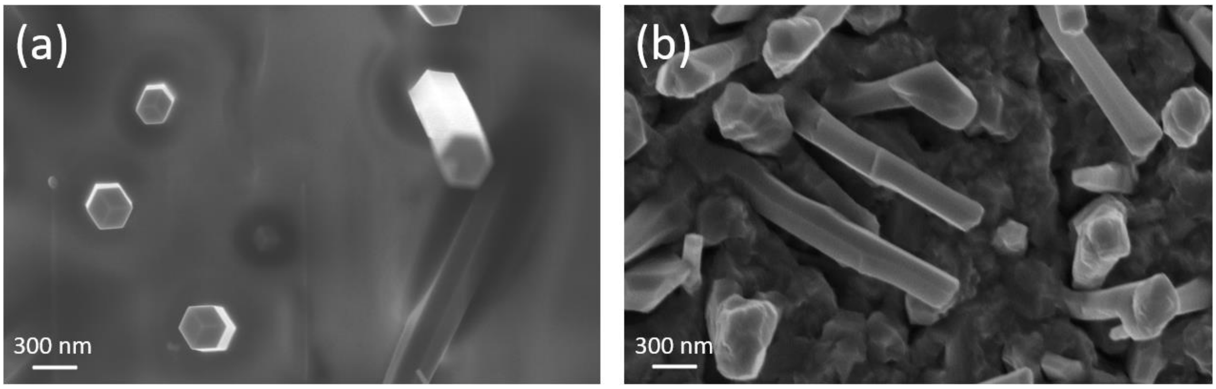

Figure S2: Top-view SEM micrograph of as-grown NWs grown using the SAE (a) and reference (b) growth methods.

S2. Threshold gain calculations

The lasing threshold in a NW Fabry-Perot cavity is determined by the following relationship

$$g_{th} = \left(\frac{1}{\Gamma}\right)\left[\alpha_i + \frac{1}{2L}\ln\left(\frac{1}{R^2}\right)\right] \qquad \text{(S1)},$$

where Γ is the mode confinement factor, describing the mode overlap with the gain medium, α_i is the propagation loss, L is the NW length and R is the reflectivity of the end facets. The mode confinement factor can be written as

$$\Gamma = \frac{c\varepsilon_0 n_r \iint_{\text{gain}} \frac{1}{2}|\boldsymbol{E}|^2 dxdy}{\iint \frac{1}{2}\text{Re}(\boldsymbol{E}\times\boldsymbol{H}^*)\cdot\hat{z}dxdy} \qquad \text{(S2)},$$

where the numerator integral is taken over the gain medium cross section, while the denominator integral is taken over all space. Here, c is the speed of light, ε_0 is the vacuum permittivity, n_r is the refractive index of the material, and **E** and **H** are the electric and magnetic fields, respectively. The parameters Γ, α_i and R can be found from finite-difference time-domain (FDTD) simulations (see Figure S3).

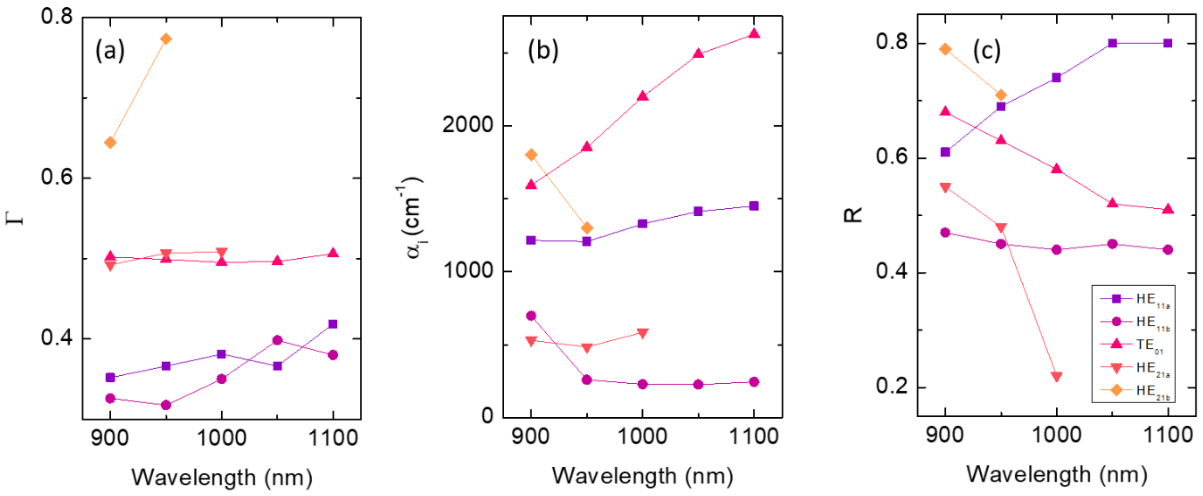

Figure S3: Simulated wavelength dependence of Γ, α_i and R for a 400 nm diameter NW.

S3. Mode analysis

It is important to single out the dominant lasing mode in the NWs. Using the FDTD results of section S2, we simulate the lasing threshold gain of the dominant waveguided modes in the NWs (Figure S4a). For wavelengths longer than 950 nm, the HE_{11a} and HE_{11b} modes have the lowest threshold gain values. Since the geometry of the reference and SAE-grown NWs studied in this work is similar, the HE_{11a} and HE_{11b} modes are expected to dominate the lasing in both samples. This can be confirmed by looking at the mode-spacing between the lasing peaks in the PL spectra (Δλ), which for Fabry-Perot cavities is given by

$$\Delta\lambda = \left(\frac{\lambda^2}{2L}\right) n_g^{-1}, \qquad (S3)$$

where λ is the light wavelength, L is the cavity length (in this case, the NW length), and n_g is the group index of refraction, determined from the FDTD calculations. Figure S4b shows the measured mode spacings for the reference (the open circles) and SAE-grown (the solid squares) NWs and the simulated dependencies (the solid lines) for the main modes using equation (S1). The majority of the NWs in both structures exhibit lasing in the HE_{11a} or HE_{11b} modes, though lasing in the HE_{21b} mode is also observed. Polarization-resolved measurements of the lasing emission support the mode assignment, exemplified by Figure S4c-e (the symbols) for the HE_{11a}, HE_{11b}, and HE_{21b} modes, respectively, along with the anticipated polarization dependence based on the FDTD calculations (the solid lines).

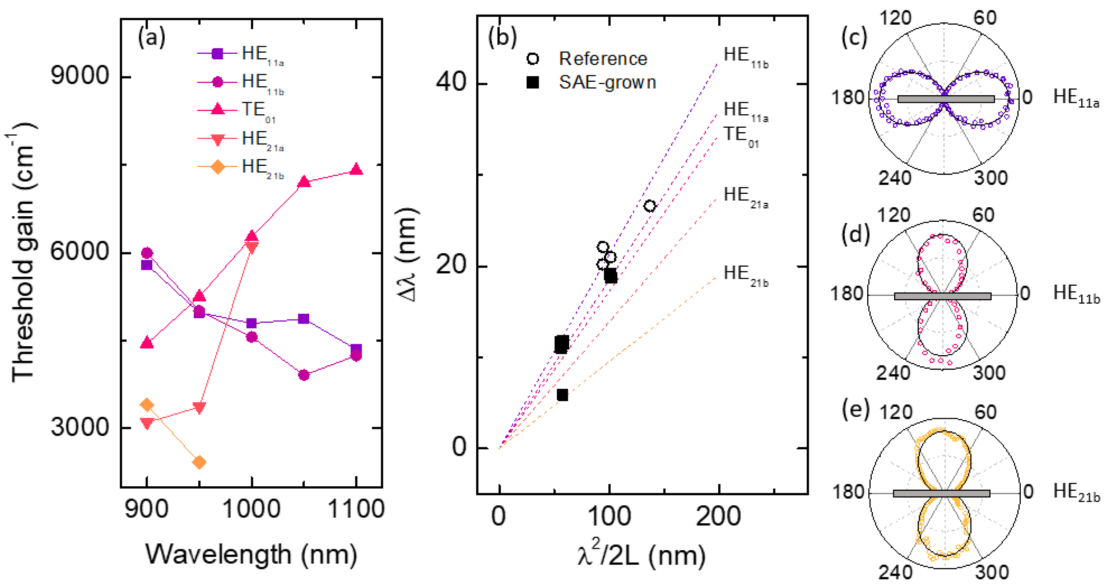

Figure S4: (a) Simulated spectral dependence of threshold gain values for the five main cavity modes of a 400 nm-thick NW at the wavelength of 960 nm. (b) Mode spacing calculated for the five main modes (the dashed lines) and measured from the reference (the open symbols) and SAE-grown (the closed symbols) structures. (c)-(e) Measured (the symbols) and calculated (the lines) polar plots of the HE_{11a}, HE_{11b}, and HE_{21b} modes, respectively. The gray bars indicate the NW orientation.

S4. Optical quality of the NWs

Figure S5 shows the averaged PL spectra acquired from ten NWs of the reference (orange) and SAE-grown (purple) samples. The total PL intensity is similar for the two samples, though the spectrum of the reference structures extends a little further into the NIR range, indicative of a higher localization energy.

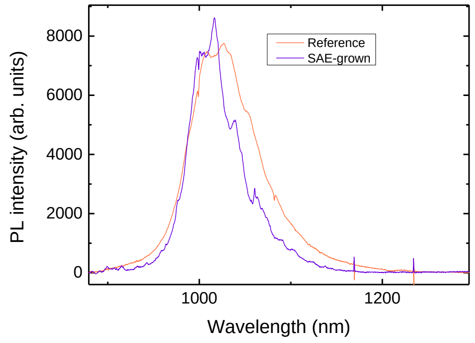

Figure S5: µPL spectra averaged over 10 SAE-grown (purple) and reference (orange) NWs. The measurements were performed using a cw-laser emitting at 800 nm wavelength, at 5 K.

By measuring the change of the PL intensity as a function of temperature (see Figure S6a and b for individual and arrays of standing NWs, respectively), we see no clear difference between the samples. This implies that rates of non-radiative recombination, which causes quenching of the PL intensity with increasing temperature, are the same for the two growth methods.

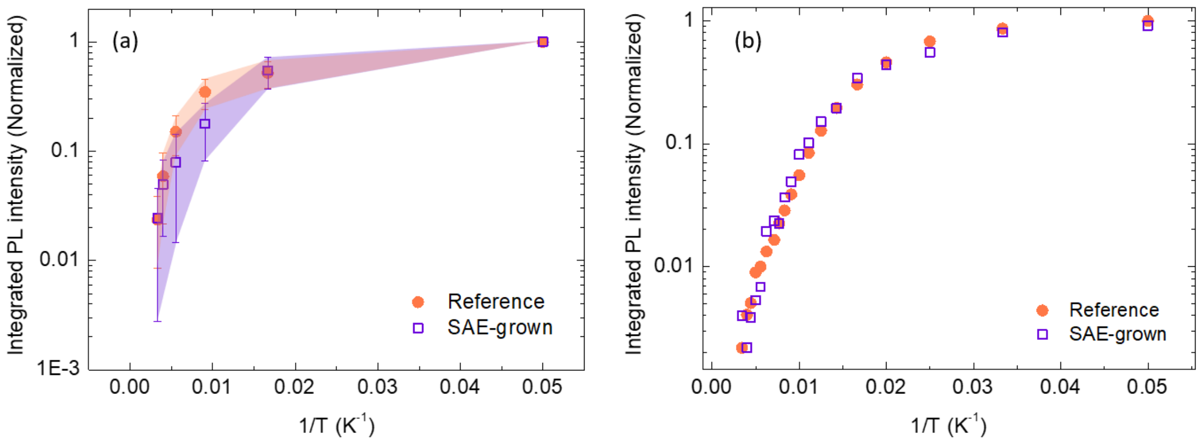

Figure S6: (a) Average integrated PL intensity acquired from 10 reference (the orange circles) and SAE-grown (the purple squares) NWs. The error bars indicate the standard deviation. (b) Integrated PL intensity acquired from reference (the orange circles) and SAE-grown (the purple squares) NW arrays. The measurements were performed using a cw-laser emitting at 800 nm wavelength.

The two samples are growth using different cooling mechanisms of the shroud, which affects the background pressure during the growth. Though the use of the higher background pressure may affect incorporation of background impurities, it does not affect the PL intensity of our NWs. As demonstrated in Figure S7, no significant difference of the PL intensity is observed for the NWs grown with a water- (black) and LN_2 - cooled (blue) shroud. Thus, in our case the cooling system of the MBE chamber did not affect the optical quality of the nanowires. In combination with the results of Figures S5 and S6, this confirms that the background pressure during the NW growth does not influence the formation of non-radiative defects and, therefore, the lasing performance of the studied NWs.

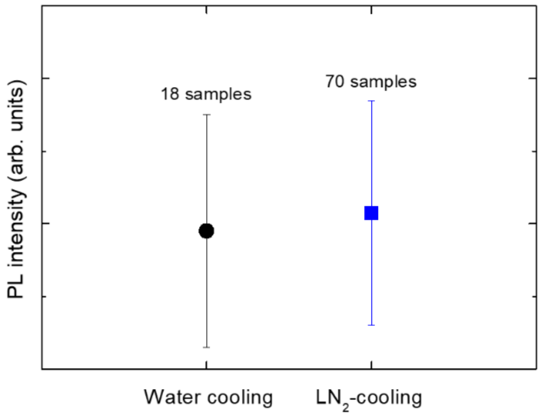

Figure S7: The average PL intensity among NW samples grown using water cooling (the black dot) and LN2 cooling (the blue square). The error bars indicate the standard deviation.

S5. Effects of the structural properties on the lasing threshold

Figure S8 compares the lasing threshold of two SAE-grown and two reference NWs of similar lengths and widths. Despite similarities in geometrical dimensions, the lasing threshold is much lower in the SAE-grown NWs.

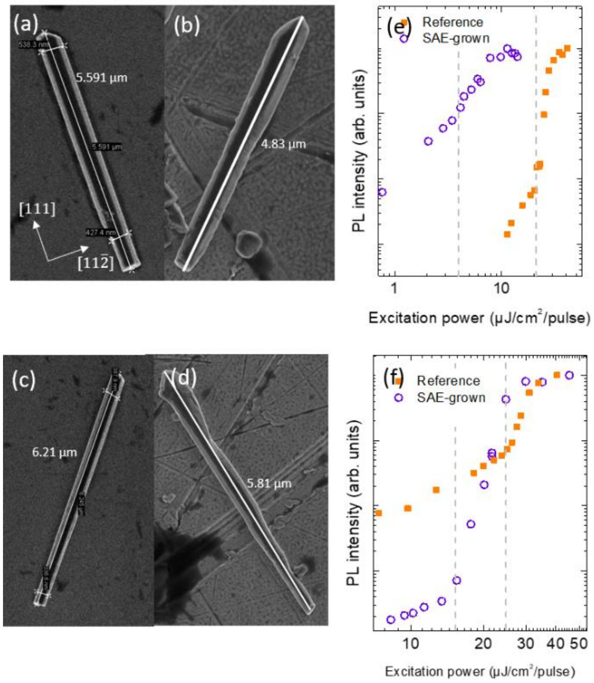

Figure S8: SEM micrographs of two SAE-grown (a, c) and two reference (b, d) NWs. (e, f) Power dependence of the PL intensity of the same NWs as shown in (a-d).

Figure S9 shows the lasing threshold vs NW length for SAE-grown (squares) and reference (circles) NWs, where no correlation between these parameters can be observed.

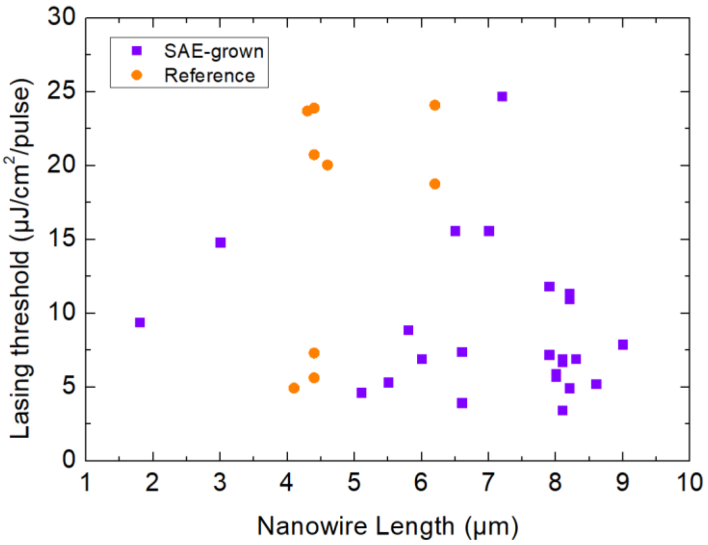

Figure S9: Lasing threshold vs. NW length, for SAE-grown (purple squares) and reference (orange circles) NWs.

From Figs S8 and S9, it is apparent that the observed improvement in the lasing performance of the SAE-grown NWs is not primarily related to their length.

S6. Localization Energy

In highly mismatched materials, such as GaNAs, the exciton localization due to alloy fluctuations can be very large. In such materials, the exciton density of states contains a low-energy tail comprising localized exciton states, located below the free-exciton energy. For energies below the band gap energy, the density of the localized states (D_{LS}) within such a band tail decays exponentially at lower energies as:

$$D_{LS} \propto e^{\frac{E}{E_0}} \qquad \text{(S4)},$$

where E is the exciton energy and E_0 is the characteristic localization energy, which is a measure of the degree of localization in the material. The localization energy of an individual NW can, therefore, be extracted from the acquired PL spectra at low excitation densities, as exemplified in Figure S10.

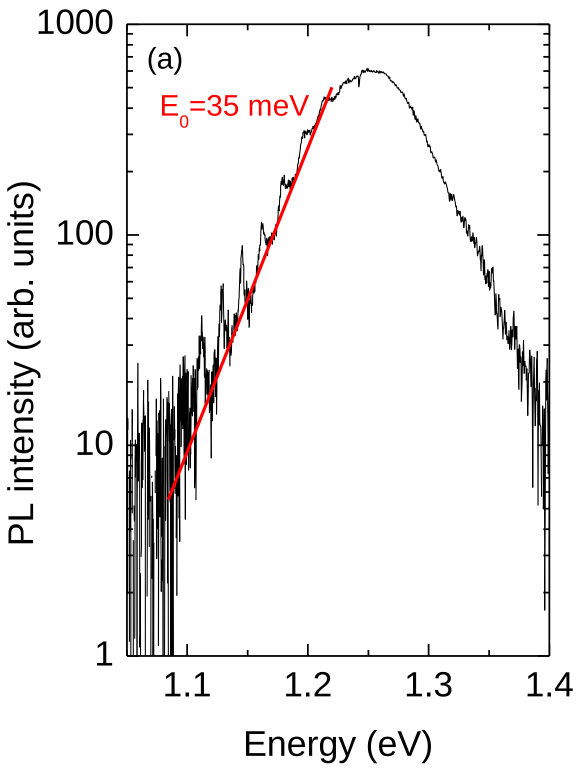

Figure S10: Exponential fit of the low-energy tail of the PL spectra from an SAE-grown (a) and reference (b) NW to extract the localization energy, E_0.

S7. Rate equation analysis

To model the recombination dynamics of the system, we use a set of rate equations (adapted from [3]), governing the concentration of free excitons (N), localized excitons (N_{LE}) and photons in the lasing photon mode (S):

$$\frac{dN}{dt} = \frac{\eta_p P}{\hbar\omega V} - \frac{N}{\tau_{sp,FE}} - CN^3 - v_g g_{\max}(N)S - \frac{N}{\tau_{\text{rlx}}}\left(1 - \frac{N_{LE}}{D_{LS}}\right) \quad \text{(S5),}$$

$$\frac{dN_{LE}}{dt} = \frac{N}{\tau_{\text{rlx}}}\left(1 - \frac{N_{LE}}{D_{LS}}\right) - \frac{N_{LE}}{\tau_{sp,LE}} \quad \text{(S6),}$$

$$\frac{dS}{dt} = \Gamma v_g\left(g_{\max}(N) - g_{th}\right)S + \Gamma\beta\left(\frac{N}{\tau_{sp,FE}} + \frac{N_{LE}}{\tau_{sp,LE}}\right) \quad \text{(S7).}$$

Here, η_p is the absorption efficiency of laser power by the NW, taken to be 1%[3], P is the excitation power, V is the NW volume calculated for a 6 µm-long NW having a 400 nm diameter, $\tau_{sp,FE}$ and $\tau_{sp,LE}$ are the spontaneous recombination lifetimes of the free and localized excitons, respectively, both taken to be approximately 3 ns,[3] C is the Auger coefficient, assumed to be 10^{-30} cm^6/s.[2] v_g and Γ are the group velocity and mode confinement factor of the mode, deduced from the FDTD calculations – see section S3. g_{th} is the threshold gain which is obtained to assure the best fit to the data of Figure 2c of the main text, τ_{rlx} is the relaxation time of free excitons into the localized states,[3] D_{LS} is the density of

localized states and β is the spontaneous emission coupling factor. $g_{max}(N)$ is the peak gain, which can be calculated from

$$g_{\max}(N) = g_0 \ln\left(\frac{N + N_s}{N_{tr} + N_s}\right) \qquad (S8),$$

where the values of g_0, N_s and N_{tr} are taken from [3]. Table S1 displays the values for the parameters used in the simulations displayed in the main paper, where D_{LS} and β are used as variables.

Table S1. The parameter values used in the rate-equation model for simulations in this work. Here, c is the speed of light.

η_p	$\hbar\omega$ (eV)	$\tau_{sp,FE}=\tau_{sp,LE}$ (ns)	C cm^6/s	v_g	N_{tr} (cm^{-3})	N_s (cm^{-3})	τ_{rlx} (1 ps)	Γ	g_{th} (cm^{-1})
1%	1.29	3	10^{-30}	c/5.4	$8\cdot10^{17}$	$1.2\cdot10^{17}$	1	0.38	4000

S8 Band gap energy estimation

Figure S11 shows the squared photoluminescence excitation (PLE) intensity acquired from the reference (the orange line) and SAE-grown (the purple line) NWs, respectively. From the PLE curves, the band gap energies may be estimated as the intersection of their tangents with the x-axis. By this process (see the black lines in the figure) we find the band gap energies of 1.21 and 1.20 eV for the SAE-grown and reference samples, respectively. Using these band gap energies, the nitrogen content can be estimated from the band-anticrossing model, where the relationship between the band gap energy and nitrogen content is given by:

$$E_g^{GaNAs} = \frac{1}{2}\left[\left(E_g^{GaAs} + E^N\right) - \sqrt{\left(E_g^{GaAs} - E^N\right)^2 + 4V^2x}\right] \qquad (S9),$$

where E_g^{GaAs} is the band gap energy of the nitrogen-free GaAs, x is the nitrogen content, E^N is the energy level of the single nitrogen impurity and the V-parameter determines the strength of the interaction between the host and nitrogen states.[4] The last two parameters have been previously estimated as 1.65 eV and 2.5 eV, respectively.[4,5] From the equation, we find that the nitrogen concentrations in the SAE-grown and reference samples are 2.2% and 2.3%, respectively.

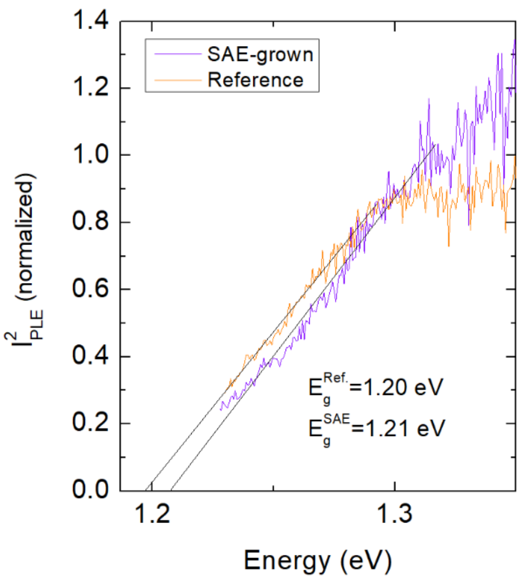

Figure S11: PLE spectra acquired from arrays of SAE-grown (the purple line) and reference (the orange line) NWs at 4 K. The black lines are the tangents of the PLE curves, which are used to estimate the band gap energy by their intersection with the x-axis.

S9 Statistical analysis

t-test

The t-test tests the null-hypothesis that the population means of two populations is equal:

$$H_0: \mu_1 = \mu_2 \tag{S10}$$

The test statistic, T, is given by

$$T = \frac{\bar{x}-\bar{y}}{\sqrt{\frac{s_x}{n}+\frac{s_y}{m}}} \tag{S11}$$

where $\bar{x}$ and $\bar{y}$ are the sample means, s_x and s_y are the pooled standard deviations and n and m are the sample sizes. From T, the p-value may be calculated as the probability of finding the given T if the null hypothesis is true. For a given significance level, α, the null hypothesis may be rejected if p<α.

Linear regression test

As a measure of the linear dependence of two variables A and B in a sample, the Pearson correlation coefficient may be used:

$$\rho(A, B) = \frac{\mathrm{cov}(A,B)}{\sigma_A \sigma_B} \tag{S12}$$

Here, cov(A,B) is the covariance of the two variables, and σ_A and σ_B are the sample standard deviations. From ρ we can calculate the p-value, which is used to test the null hypothesis that the ρ of the population is 0:

$$H_0: \rho_P = 0 \tag{S13}$$

To determine how well the linear model can explain the variance in the data, the coefficient of determination, R^2 ($=\rho^2$), can be used.

S10 Influence of the β-factor on the lasing threshold

The β factor affects the photon density in the lasing mode, which relates to the rate of stimulated emission. However, the main influence of the lasing threshold stems from the concentration of free excitons, N, which is not directly related to β but depends on non-radiative recombination (which is the same in both structures) and also the density of localized states. By carefully evaluating the simulation results, we note that with the simulation parameters used in this manuscript (see Table S1 of section S7), changes of β within the range of 0.01-0.05 do not significantly affect the lasing threshold. This is shown in Figure S12, which shows the power dependence of the lasing mode photon density, S, simulated using the parameters of Table S1 of section S7, a localized state density of $7 \cdot 10^{18}$ cm^{-3}, and β varying between 0.01 and 0.05. In all simulations, the lasing threshold is estimated to be ~170 mW. We also note that similar results showing a lasing threshold independent of β have been previously reported.[5]

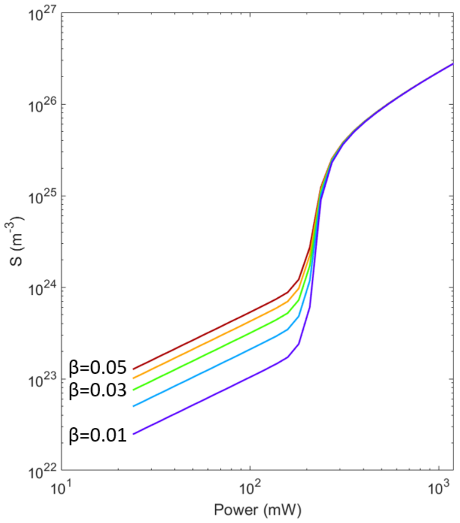

Figure S12. Simulated photon density, S, as a function of excitation power density obtained assuming a fixed density of the localized states ($7 \cdot 10^{18}$ cm^{-3}) for several parameters β relevant to this work.

References

[1] M. Yukimune, R. Fujiwara, T. Mita, N. Tsuda, J. Natsui, Y. Shimizu, M. Jansson, R. Balagula, W. M. Chen, I. A. Buyanova, F. Ishikawa, *Nanotechnology* **2019**, *30*, 244002.

[2] D. Saxena, S. Mokkapati, P. Parkinson, N. Jiang, Q. Gao, H. H. Tan, C. Jagadish, *Nat. Photonics* **2013**, *7*, 963.

[3] S. Chen, M. Yukimune, R. Fujiwara, F. Ishikawa, W. M. Chen, I. A. Buyanova, *Nano Lett.*

2019, *19*, 885.

[4] W. Shan, K. M. Yu, W. Walukiewicz, J. Wu, J. W. Ager III, E. E. Haller, *J. Phys.: Condens. Matter* **2004,** *16*, S3355.

[5] I. Suemune, K. Uesugi, W. Walukiewicz, *Appl. Phys. Lett*. **2000,** *77*, 3021.

[6] B. Mayer, L. Janker, B. Loitsch, J. Treu, T. Kostenbader, S. Lichtmanneker, T. Reichert, S. Morkötter, M. Kaniber, G. Abstreiter, G. Koblmüller, J. J. Finley, *Nano Lett.* **2016**, *16*, 152.
